# An Automated Data Fusion-Based Gear Faults Classification Framework in Rotating Machines

**DOI:** 10.3390/s21092957

**Published:** 2021-04-23

**Authors:** Ruifeng Cao, Akilu Yunusa-Kaltungo

**Affiliations:** Department of Mechanical, Aerospace and Civil Engineering, University of Manchester, Manchester M13 9PL, UK; ruifeng.cao@manchester.ac.uk

**Keywords:** spectrum energy, artificial neural network, data fusion, composite spectrum, vibration-based condition monitoring, rotating machines

## Abstract

The feasibility and usefulness of frequency domain fusion of data from multiple vibration sensors installed on typical industrial rotating machines, based on coherent composite spectrum (CCS) as well as poly-coherent composite spectrum (pCCS) techniques, have been well-iterated by earlier studies. However, all previous endeavours have been limited to rotor faults, thereby raising questions about the proficiency of the approach for classifying faults related to other critical rotating machine components such as gearboxes. Besides the restriction in scope of the founding CCS and pCCS studies on rotor-related faults, their diagnosis approach was manually implemented, which could be unrealistic when faced with routine condition monitoring of multi-component industrial rotating machines, which often entails high-frequency sampling at multiple locations. In order to alleviate these challenges, this paper introduced an automated framework that encompassed feature generation through CCS, data dimensionality reduction through principal component analysis (PCA), and faults classification using artificial neural network (ANN). The outcomes of the automated approach are a set of visualised decision maps representing individually simulated scenarios, which simplifies and illustrates the decision rules of the faults characterisation framework. Additionally, the proposed approach minimises diagnosis-related downtime by allowing asset operators to easily identify anomalies at their incipient stages without necessarily possessing vibration monitoring expertise. Building upon the encouraging results obtained from the preceding part of this approach that was limited to well-known rotor-related faults, the proposed framework was significantly extended to include experimental and open-source gear fault data. The results show that in addition to early established rotor-related faults classification, the approach described here can also effectively and automatically classify gearbox faults, thereby improving the robustness.

## 1. Introduction

Since the rise of machines and consequent industrial revolutions, rotating machines have become an integral and inevitable asset within virtually all industrial setups, irrespective of the sector. This is mainly due to their versatility and ability to adapt to the incredibly harsh operating environments that prevail in most industries. Components such as electric motors [1,2,3,4] and gearboxes are integral to the functioning of numerous industrial rotating systems and are often envisaged to be robust enough to withstand highly dynamic operations.

However, despite their often-resilient designs and configurations, gearboxes still experience failures, some of which have direct devastating consequences on income, environment, and human safety. Although the value of gearboxes has never been undermined, their criticality (especially due to their contributions to failure rates) to alternative energy systems, however, has further increased the intensity of the scrutiny they have received in recent times. For instance, the study by Spinato et al. [5] highlighted that wind turbine (WT) gearboxes account for the highest mean-time-to-repair among all onshore sub-assemblies. Similarly, Gray and Watson [6] also highlighted that as much as 33% of total operational downtime of energy generation systems can be attributable to their gearboxes. The findings from these studies [5,6] have also been corroborated by regional surveys and other technical reports. For instance, 3-year WT operational data from offshore wind farms in Netherlands stated that gearbox failures can account for more than 55% of total annual downtime, which sometimes corresponds to over 52% of energy not served [7]. Feng et al. [8] also conducted a similar survey for selected wind farms in the United Kingdom and reported nearly identical findings.

In general, incessant failure of the gearboxes of industrial systems are often attributed to inaccurate estimation of actual operating loads, unforeseen changes to loading conditions during operations, faulty component design, and/or inaccurate repair/replace decisions [9]. Traditionally, maintenance interventions (mainly repair and replace) have served as remedies to such failures since the amount of consideration allotted to downtime was insignificant in the past. In contrast to such times, modern-day operations are very lean and mainly customer-oriented, owing to fierce global competitions. This is perhaps the reason for the surge in the popularity of predictive and condition-based maintenance (CBM) strategies [10,11,12], whereby industrial assets dictate the frequency of maintenance interventions. Just as gearboxes have earned themselves the status of inevitability within most industrial operations, vibration monitoring (VM) [13,14,15] is arguably one of the most widely applied CBM techniques owing to the established fact that all structures (static or rotating) exhibit their own peculiar individual dynamic characteristics. The fundamental premise of VM is to adequately understand, track, and determine the trend of these characteristics for individual critical assets, so as to determine deviations at incipient stages before the occurrence of catastrophic failures. Despite the huge successes recorded with well-established VM techniques in time [16,17], frequency [18], and time–frequency [19] domains, the rigour often associated with individualised synthesis of large volumes of data acquired from each measurement location on a typical rotating machine can prolong decision-making, which may lead to fatal consequences when dealing with critical safety systems. To further compound this problem, most modern-day industrial rotating machines are multi-component (e.g., gears, bearings, drive belts, rotors, electric motors, couplings, etc.) and multi-state (e.g., speed and load variations), thereby requiring a holistic approach to VM.

In order to alleviate this limitation and better optimise conventional rotating machine VM approaches, research endeavours over the past few decades have been exploring mechanisms through which VM information can be harmonised into single but representative frameworks. Such approaches are generally referred to as data fusion, information fusion, or hybridisation. In general, data fusion involves the combination of data and information from different sources to obtain enhanced accuracy that may not be achievable from a single source. This approach has the potential to overcome some of the real-life problems that have plagued the use of VM for incipient fault detection and diagnosis. Data fusion can occur at different stages of a typical faults classification process, with sensor, feature, and decision levels being the most common stages. At the sensor or data acquisition level, data from various commensurate sensors are directly fused, after which the most representative features are used to take decisions [20,21,22,23,24]. At the feature level, individual sensors are used to acquire data separately, and the required feature vectors extracted. The feature vectors are eventually fused together and form the basis for decision-making of operational state classification [25,26,27]. Finally, the decision-level fusion approach harmonises the estimated decisions that have been separately drawn from the feature vectors of individual sensors [28,29].

Generally, machinery fault diagnosis approaches that embed machine learning techniques within them usually entail two key phases, namely feature extraction and fault identification. The feature vectors generated during the feature extraction phase are usually applied as inputs in the chosen machine learning technique(s) for the fault identification phase [30]. The fundamental essence of fault identification is to distinguish healthy from faulty machine conditions, based on the extracted features, which is also equivalent to the mapping of information from the feature space to the fault space [30]. Classifiers and statistical learning methods, such as k-nearest neighbour (k-NN) algorithms, Bayesian classifier, support vector machine (SVM), and artificial neural network (ANN) have been widely utilised in structural health monitoring systems of rotating machinery [30].

Kalman filtering [31], weighted average [32], algebraic functions [33], Bayesian estimators, nonlinear system fusion, and adaptive observers [34] are some of the earlier and well-established traditional sensor fusion approaches. Despite the usefulness of the academic research advancements achieved through these techniques, their mathematical intrusiveness could be a reason for their limited application for real-life rotating machine faults classification. For instance, Bayesian estimators are popular; the processing frameworks for dynamic models that are nonlinear often entail some multi-dimensional integrals that are often analytically intractable, thereby leading to estimate difficulties. In addition, the generated outputs are sometimes generic and associated with multimodality, asymmetries, and discontinuities [35]. With regards to Kalman filtering, linearised transformations are only proficient when error propagations can be estimated using a linear function; otherwise, the generated linearised outputs are negatively impacted and lead to complete divergence [36]. Adaptive observers can be very power and accurate when dealing with continuous time domain signals, but their effectiveness dwindles under low control and switching frequency, which is often an attribute of nonlinearities of several rotating machine components, especially gear systems [37]. Moreover, each of these classes of techniques [31,32,33,34] are often focussed on a single stage fusion as well as applied to a single machine component, thereby increasing computational complexity and, in turn, further complicating an already tedious faults classification process. On the contrary, the recently developed composite coherent spectrum (CCS) [38] and poly-coherent composite spectrum (pCCS) [38,39,40,41] significantly reduce computational stages associated with data fusion since it embeds both sensor-level and feature-level fusion into a single framework.

While previous studies on CCS [38] and pCCS [38,39,40,41] have yielded encouraging outcomes, they have only been applied to rotor-related machine faults (mainly misalignment, rub, crack, and bow), which does not adequately represent the multi-component configuration of most modern-day rotating machines. Besides the restriction of scope of the founding CCS and pCCS studies with respect to rotor-related faults, their diagnosis approach is manually implemented, which could be unrealistic when faced with routine condition monitoring of multi-component industrial rotating machines, which often entails high-frequency sampling at multiple locations. The automated framework applied here was recently presented by Yunusa-Kaltungo and Cao [42] to help address the laborious nature of manual faults classification of CCS. Although the framework encompassed initial feature generation through CCS data fusion, data dimensionality reduction via principal component analysis (PCA) and subsequent faults classification was achieved using several machine learning techniques, including ANN, SVM, k-NN, etc. The outcomes of the automated approach are usually a set of visualised decision maps representing individually simulated scenarios, which simplifies as well as minimises diagnosis-related downtime by allowing asset operators to easily identify anomalies at their incipient stages without necessarily possessing vibration monitoring expertise. Additionally, comparisons between other VM techniques indicated that CCS has significant advantages as a feature extraction method, owing to its ability to greatly reduce potential complexities that are sometimes associated with the machine learning input datasets. The results obtained from the study [42] were encouraging and showed that ANN was most compatible with CCS. However, the study was also limited by its application to only rotor-related faults.

This study adequately extends previous works on CCS and pCCS based on two main premises. Firstly, it establishes a framework by which the diagnosis of multiple classes of rotating machine faults can be automated through machine learning algorithms. The second major contribution of this study is that it significantly builds upon the encouraging results obtained from the preceding part of this approach that was limited to well-known rotor-related faults [42] by incorporating gearbox fault detection into a single framework. Hence, the extension provided here now considers an entirely different and unique class of rotating machine components—the gearbox, so as to complement earlier findings and ascertain robustness. Additionally, the proposed approach is primarily based on tools and features that are universally established across academia and industry (especially amplitude spectrum), thereby easing the transfer of theoretical knowledge into practice.

To accomplish this, the paper initially compares the proficiency of its approach to that of earlier related approaches in Section 2, after which a brief theoretical overview of the proposed framework is provided in Section 3 such that the current paper can be fully comprehended without the need to consult earlier articles. Section 4 provides full details of the experimental designs, with particular emphasis on the experimental rig configuration, types of machine operating conditions simulated, technical specifications of instruments, and signal processing parameters. Previous studies [42] have already recommended several rotor fault detection features, but it is uncertain that all of such features will adequately support the computational effectiveness of the current study. Therefore, in Section 5, the performance of relevant features is initially examined, after which the most influential features are then identified. In Section 6, the results of faults classification based on the proposed approach are presented as well as explanations of the implications of the findings for VM of rotating machines (in this case, gearbox faults). Section 7 provides the validation of the applied method with independent public datasets. Finally, Section 8 concludes the study and highlights possible future directions.

## 2. Comparison with Closely Related Works

The study of fault diagnosis in rotating machines is well-established and continues to generate spates of useful but sometimes closely related outputs in some cases, which makes it imperative to compare and contrast to identify niche areas. Therefore, this section is based on a comparative analysis between closely related approaches in fault diagnosis of rotating machines and the current study, so as to clearly highlight areas of potential interface, overlap, variation, limitation, and superiority. In order to better show the advantages of the proposed method and point out future research directions, Table 1 provides comparisons with other recent studies in a similar area. The main criteria used for comparison are the data types, classification algorithm(s), application of data fusion, and fault classes considered.

Yunusa-Kaltungo et al. developed CCS [38] and pCCS [38,39,40,41], which significantly rationalised computational stages associated with fault diagnosis through data fusion by embedding both sensor- and feature-level fusion into a single framework. However, their application has been limited to rotor-related machine faults and entail manual classification, which will increase the downtime related to fault diagnosis. This limitation led to the proposal of an automated framework [42] that still used features generated via CCS but further involved data dimensionality reduction by PCA and eventual machine learning-based faults classification. The outcomes were very encouraging especially that the study [42] exposed the compatibility of ANN with CCS but study was again confined to rotor-related faults thereby not all encompassing.

Cao et al. [43] developed a deep transfer learning approach based on a convolutional neural network (CNN) algorithm, and their study advocated the suitability of the approach for deep feature extraction and gear fault diagnosis. Similarly, Shao et al. [44] developed a CNN-based deep transfer learning framework for mechanical fault diagnosis and classification, while Soualhi et al. [45] proposed a health indicator fed into an adaptive neuro-fuzzy inference system (ANFIS) to detect the state of health of a typical system and then diagnose sources of anomalies. The data collected through this method are electrical signals, mainly current signals as opposed to mechanical signals such as vibrations for non-invasive benefits, since the current, voltage, or power sensors that are already integrated into the control systems of electrical machines can be used. Azamfar et al. [46] developed a novel multi-sensor data fusion methodology based on 2-D CNN for gearboxes fault diagnosis using motor current signature analysis. Zhang et al. [47] proposed a novel unsupervised learning algorithm named fast intrinsic component filtering (FICF) for the fault diagnosis of rotating machinery. These studies have no doubt enhanced the knowledge around the fault diagnosis of rotating machines. However, they are limited by either focus on the class of singular faults (e.g., rotor faults or gear faults or bearing faults alone) which implies that alternative approaches will need to be considered for other fault classes, thereby increasing rigour and downtime or computational intensiveness of CNN-based approaches. Table 1 provides more targeted merits, demerits, and coverage of individual study classes.

## 3. Theoretical Overview of the Approach

### 3.1. Mathematical Representation

**Figure 1:**
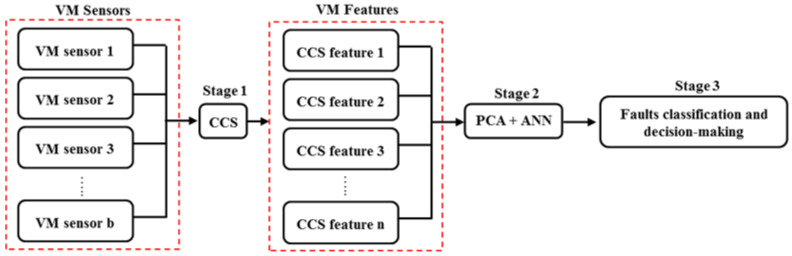
Schematic representation of the faults classification process.

It is vital to highlight that full details of the automated faults classification framework applied here have been provided in the preceding article that focussed on rotor-related faults [42]. However, the provision of high-level description, here and again, was adjudged useful, so as to allow this second part to be comprehensible as a standalone section. The process commences with the use of the CCS approach [38] to fuse amplitude spectra computed from the time domain datasets acquired by individual VM sensors installed on the rotating machine. The mathematical representation of the CCS process is iterated in Equations (1)–(3) [38,39,40,41]. The fundamental rationale behind the CCS is that it eliminates downtime associated with routine VM processes, especially when such monitoring returns a no-fault result from the studied machine. During such VM processes, technicians are required to analyse data from all measurement locations on the machine, but this process is minimised by the CCS as only one spectrum needs to be routinely observed. The only instance that would warrant analysis of individual spectra is when a deviation from the single CCS is observed. This stage of the fusion is referred to as Stage 1 in Figure 1 and further explained thus:

If the number of measurement points on a particular rotating machine is b, each of which is furnished with a VM sensor, then the vibration signals acquired from individual sensor can be divided into $n_{s}$ equal-length segments. The coherent cross-power spectral density of the signals from the pth and (p+1)th measurement points at a frequency $f_{h}$ can be defined as:

(1) $$S_{x_{p}\gamma_{p\left(p+1\right)}^{2}x_{p+1}}^{r}\left(f_{h}\right)=\left[X_{p}^{r}\left(f_{h}\right)\gamma_{p\left(p+1\right)}^{2}\left(f_{h}\right)X_{p+1}^{r^{*}}\left(f_{h}\right)\right]$$

where $X_{p}^{r}\left(f_{h}\right)$ is the discrete Fourier transform (FT) of the rth segment of the signal $x_{p}$, and $X_{p}^{r^{*}}\left(f_{h}\right)$ is its complex conjugate, for p=1, 2,…,b−1. $\gamma_{p\left(p+1\right)}^{2}\left(f_{h}\right)$ is the coherence of the signals $x_{p}$ and $x_{p+1}$ for background noise suppression.

Hence, each of the rth segments from each signal can be fused into a single component, $X_{CCS}^{r}\left(f_{h}\right)$, thus:

(2) $$X_{CCS}^{r}\left(f_{h}\right)=\sqrt{{\left(\begin{array}{c} S_{x_{1}\gamma_{12}^{2}x_{2}}^{r}\left(f_{h}\right)S_{x_{2}\gamma_{23}^{2}x_{3}}^{r}\left(f_{h}\right)\\ \ldots S_{x_{\left(b-1\right)}\gamma_{\left(b-1\right)b}^{2}x_{b}}^{r}\left(f_{h}\right) \end{array}\right)}^{\frac{1}{\left(b-1\right)}}}$$

The CCS for the entire machine can then be calculated as:

(3) $$S_{CCS}\left(f_{h}\right)=\frac{\sum_{r=1}^{n_{s}}X_{CCS}^{r}\left(f_{h}\right)X_{CCS}^{r^{*}}\left(f_{h}\right)}{n_{s}}$$

The $S_{CCS}\left(f_{h}\right)$ is a sequence of complex numbers that enables the estimation of the amplitude spectrum of the CCS according to Equation (4):

(4) $$A_{CCS}\left(f_{h}\right)=\left|\frac{2}{N}S_{CCS}\left(f_{h}\right)\right|\;\;\;\;\;\;\;\;\;\;\left(h=1,\;2,\ldots ,N/2\right)$$

In addition to the earlier CCS harmonic amplitudes that only offer a single-point value of differentiation that could be similar for several harmonics, here, we also consider the spectral energy that can be estimated according to Equation (5).

For a typical $A_{CCS}\left(f_{h}\right)$ computed as per Equation (4) at a frequency $f_{h}$, where $f_{h}=\left(h-1\right)df,h=1,2,\ldots ,N/2$, N is the number of data points and df is the frequency resolution, the SE between the selected harmonics at intervals of df can be defined as:

(5) $$A_{SE}\left(f_{h}\right)=\overset{h+10}{\underset{i=h-10}{\sum }}A_{CCS}\left(f_{i}\right)\times df\;\left(h=11,12,\cdots ,N/2-10\right)$$

In order to analyse the gear fault, in addition to considering the harmonics of rotating speeds of gears and shafts, the harmonics of the gear mesh frequency (GMF) should also be included in CCS calculation because sidebands around the GMF and its harmonics contain information on gearbox faults [48]. The GMF can be calculated by:

(6) $$f_{GMF}=z_{t}\times n_{R}$$

where $z_{t}$ is the number of teeth on pinion, and $n_{R}$ is the rotating speed of the pinion.

Once the CCS harmonic amplitudes and their corresponding SEs of interest (depending on the fault types considered, e.g., low frequency for rotor-related and higher frequency for gear faults) have been obtained, Stage 2 of fusion involves their standardisation, dimensionality reduction, and harmonisation based on PCA [49,50,51] and ANN [52,53,54]. The computational steps required for Stage 2 are described by Equations (7)–(11).

Owing to the variations in the amplitude ranges that may be associated with the diagnosis of a complex multi-component system such as that considered here (e.g., shaft and gear mesh frequency harmonic amplitudes), dimensionality reduction through PCA would require some prior standardisation of the input data A. In the matrix $A\in R^{m\times n}$, m is the number of samples and n is the number of features (dimensions), $a_{ij}$ represents typical elements of the matrix, while $x_{ij}$ is a corresponding element of the standardised matrix X.

The element $x_{ij}$ of the standardised matrix X is defined as:

(7) $$x_{ij}=\frac{\left(a_{ij}-{\bar{A}}_{j}\right)}{S_{j}}$$

where ${\bar{A}}_{j}$ is the sample mean of the elements of the jth column of matrix A, and $S_{j}$ is the sample standard deviation of the jth column of A, which is mathematically represented as:

(8) $$S_{j}=\sqrt{\frac{\sum_{i=1}^{m}{\left(a_{ij}-{\bar{A}}_{j}\right)}^{2}}{m-1}}$$

The computation of PCs of X reduces to the solution of an eigenvalue–eigenvector problem:

(9) $$C_{X}V=V\Lambda$$

where $C_{X}$ is the covariance matrix of X, and V is the orthogonal matrix whose jth column is the jth eigenvector of $C_{X}$, corresponding to the jth largest eigenvalue of $C_{X}$ which is the jth diagonal element of the diagonal matrix Λ.

The columns of the matrix $V\in R^{n\times n}$ are orthogonal unit vectors and are referred to as the right singular vectors of X.

The calculation of the score matrix (result) $T\in R^{m\times n}$ for a PCA can be mathematically represented as:

(10) T=XV

After dimensionality reduction and selection of the PCs combination that offers the highest representation, ANN is then used to classify the different experimentally simulated machine conditions as per Equation (11) [42]:

(11) $$y=f\left(W^{T}x\right)=f\left(\overset{N}{\underset{i=1}{\sum }}W_{i}x_{i}+b\right)$$

where f is the activation function, W are the weights and b is the scalar bias term.

### 3.2. Operational Description of The Approach

In this section, we provide a step-by-step description of the individual operational stages of the applied approach, so as to foster better understanding.

#### 3.2.1. Training Steps

- Feature extraction
  Obtain the CCS harmonic amplitudes and/or GMF harmonic amplitudes as well as their corresponding SEs for numbers of segment averages from a dataset with known health conditions. This provides the input data matrix $A\in R^{m\times n}$, where m is the number of samples (averages) and n is the number of features (dimensions).
- PCA application
  Compute ${\bar{A}}_{j}$, $S_{j}$, T, V as stipulated by Equations (7)–(10).
- Dimensionality reduction
  Here, we consider the L largest singular values to obtain the truncated score matrix $T_{L}\in R^{m\times L}$, where PC_1_ is the first column of $T_{L}$, PC_2_ is the second column of $T_{L}$, and so on.
- Training
  Use $T_{L}$ as the input of the ANN model. Train the model with labels (individual health conditions) so as to obtain the classifier as depicted by Equation (12):
  (12) $$z=f\left(x_{1},\;x_{2},\ldots ,\;x_{n}\right)$$
  In Equation (12), PC_1_ is equivalent to $x_{1}$, PC_2_ is equivalent to $x_{2}$, and PC_n_ is equivalent to $x_{n}$. Additionally, z denotes the resultant class of machine health condition.

#### 3.2.2. Automatic Classification Steps

- Feature extraction
  Obtain the CCS harmonic amplitudes and/or GMF harmonic amplitudes and their corresponding SEs for a new dataset that does not possess any labels so as to obtain the input data matrix $B\in R^{1\times n}$, where n is the number of features (dimensions) and $b_{ij}$ represents a typical element of the matrix B.
- Linear transform
  Project the data matrix B into the same linear space as the PCA obtained from the training steps. The element $y_{ij}$ of the transformed matrix $Y\in R^{1\times n}$ is defined as:
  (13) $$y_{ij}=\frac{\left(b_{ij}-{\bar{A}}_{j}\right)}{S_{j}}$$
  Similarly, the transformed score matrix $S\in R^{1\times n}$ is defined as:
  (14) S=YV
- Dimensionality reduction
  By considering only the L largest singular values, we obtain the transformed truncated score matrix $S_{L}\in R^{1\times L}$, where PC_1_ is $s_{11}$, PC_2_ is $s_{12}$, and so on.
- Classification
  With $S_{L}$ as the input to the trained classifier $z=f\left(x_{1},\;x_{2},\cdots ,\;x_{3}\right)$, we can obtain the health condition classification result $z=f\left(s_{11},\;s_{12},\cdots ,\;s_{1n}\right)$.

After training, by computing just 3 equations, we can determine the health condition from the CCS of a new vibration dataset. This operation will be automatically performed for all steps.

## 4. Experimental Design and Data Acquisition

Various operation conditions (mainly faults and speed variability) usually associated with typical industrial rotating machines were experimentally simulated on a laboratory scale rig, after which vibration datasets were acquired. This section offers full details of the experiments used to generate the VM datasets used in this study.

### 4.1. Rig Characteristics

**Figure 2:**
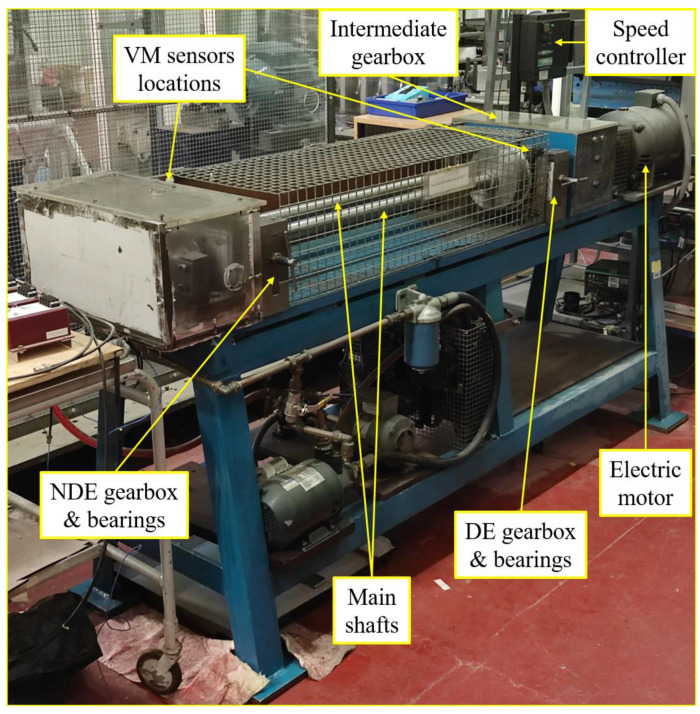
Experimental rig.

The rig used for this experiment is a multi-component rotating machine with two main rotors coupled together by two helical-geared gearboxes. Rotational force to the entire rig is provided by a 2HP electric motor that runs at a maximum speed of 3600 RPM. The electric motor shaft is coupled to the drive end (DE) gearbox through a belt pulley system. The driven pulley is directly connected to the drive shaft of the intermediate gearbox through a stepped shaft, which then transmits motion to its driven shaft via a pair of helical gears. Finally, the driven shaft of the intermediate gearbox then serves as the drive shaft for the DE gearbox through another set of helical gears. The main structure of the rig is supported by five bearings (2 bearings for each shaft and an additional bearing for the driven pulley). The rig is fitted with a lubricating system that comprised of a pump, filter, radiator, and sump for oil circulation and cooling of DE and intermediate gearboxes. The rig rotation is regulated through a variable frequency drive. All of the rotating components of the experimental rig are covered by mesh to prevent injury due to entrapment. Figure 2 and Table 2 respectively provide an image of the experimental rig and the technical specifications of its main components.

### 4.2. Instrumentation

VM data were acquired through the aid of three accelerometers (one installed on bearings near each of the three gearboxes). The raw signals from the accelerometers pass through a signal condition that also powers the accelerometers, then to an analogue-to-digital converter (ADC). Table 3 provides a summary of the technical specifications of the main instrumentation.

### 4.3. Seeded Operating Scenarios

**Figure 3:**
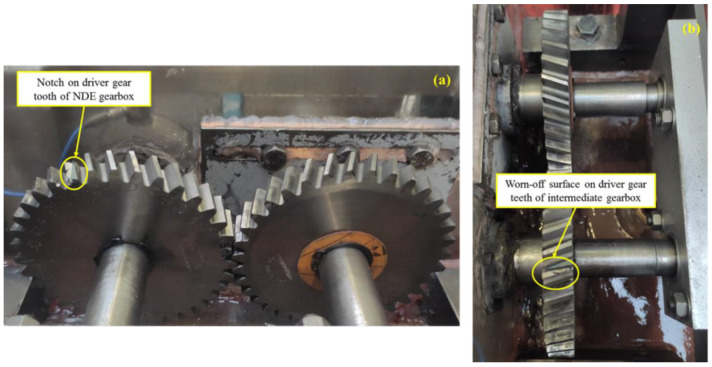
Experimentally simulated cases: (**a**) notch on driver gear tooth; (**b**) worn-off surfaces on driver gear teeth.

**Figure 4:**
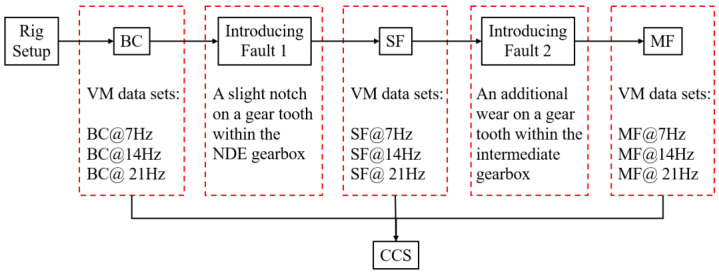
Experimental flowchart.

Considering that this was an existing multi-component rig without full knowledge of its state of health, the initial case that contained no seeded fault was termed the baseline case (BC). However, the amplitude spectra generated from the data obtained under BC displayed significantly high amplitudes at several harmonics of the machine speeds, which was adjudged to be due to inherent misalignment and unbalance faults. Therefore, BC can be classified as exhibiting rotor-related anomalies. The other two cases are the single fault (SF) and multiple fault (MF) cases. As shown in Figure 3, the SF case was simulated by introducing a slight notch on a gear tooth within the non-drive end (NDE) gearbox while the MF case was a combination of SF and additional wear on a gear tooth within the intermediate gearbox. Under all cases, VM data were collected at three distinct machine speeds (i.e., 7, 14, and 21 Hz), thereby yielding a total of nine experimentally simulated operating scenarios. In this study, a scenario represents one combination of case and speed (e.g., VM datasets for BC @ 7 Hz). The experimental flow is shown in Figure 4.

### 4.4. Data Acquisition and Signal Processing Parameters

For each of the scenarios described in Section 4.3, 3 VM datasets were acquired for approximately 120 s (totally 27 VM datasets in 9 scenarios). Here, two additional datasets are collected in each scenario to confirm that there are no anomalies in the experimental data. During spectrum and CCS calculation, the signal processing parameters used were 10,000 Hz sampling frequency ($f_{s}$), 80% segment overlap, 0.5 Hz frequency resolution (df), 448 number of segment averages, 20,000 as the number of FT data points (N), and Hanning window.

## 5. Feature Selection and Optimisation

**Figure 5:**
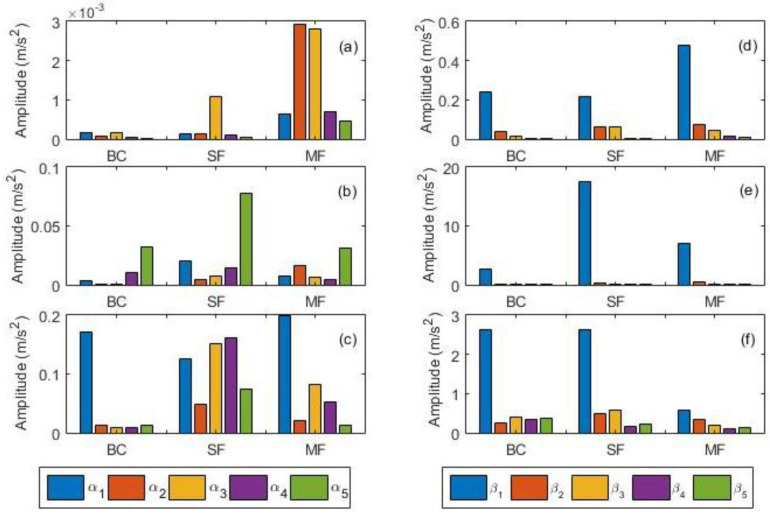
Harmonic distributions for all cases at all speeds: (**a**) rotor-related harmonics at 7 Hz; (**b**) rotor-related harmonics at 14 Hz; (**c**) rotor-related harmonics at 21 Hz; (**d**) gear mesh harmonics at 7 Hz; (**e**) gear mesh harmonics at 14 Hz; (**f**) gear mesh harmonics at 21 Hz.

**Figure 6:**
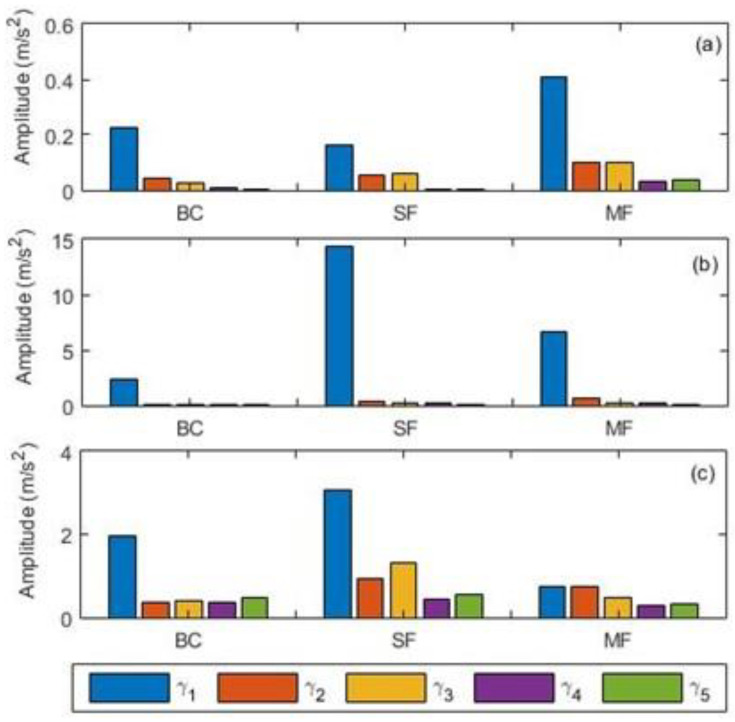
Spectral energy (SE) based gear mesh frequencies harmonics: (**a**) 7 Hz; (**b**) 14 Hz; (**c**) 21 Hz.

A typical VM process of rotating machines is usually associated with the generation of various features, especially when dealing with those characterised by multiple components. The fundamental objective of CCS data fusion approach is to rationalise data such that the VM of rotating machines can be simplified. Since the faults considered here are rotor- and gear-related, three sets of features (1^st^–5^th^ harmonics of shaft speed and 1^st^–5^th^ harmonics of gear mesh frequency) were extracted after computing the CCS as per Equations (1)–(4). Owing to the high energy contents of typical GMFs, the SE of the resultant CCS was also computed as per Equation (5) so as to observe its performance as a feature. Figure 5 shows the amplitude distributions of shaft (α_1_–α_5_) and GMF (β_1_–β_5_) features across all scenarios, where it can be seen that β_1_ was the most consistently dominant feature at all speeds and the patterns of other features were inconsistent across different scenarios. It can also be observed that BC, at all speeds, contained a prominent β_1_ feature, which is unsurprising due to the existence of inherent gears/shaft misalignment and shaft unbalance. Although the selected shaft harmonic features (α_1_–α_5_) in Figure 5 were observable for all scenarios, their distribution is similarly inconsistent. With regards to harmonic distribution, the SE-based GMFs (denoted by γ_1_–γ_5_ in Figure 6) exhibited very similar trends, except for slightly higher amplitudes of higher harmonics at 21 Hz (Figure 6). This therefore implies that while all selected features are appropriate for identifying the presence of anomalies, reliance on such features alone for fault characterisation and separation is nearly impossible due to the identified inconsistencies for different scenarios. The preliminary performance comparisons performed here aided and formed the basis for the selection of the most influential features, which then formed the basis for the next stage of the faults classification framework.

## 6. Classification Results and Their Implications

Having established the most influential features for both shaft/rotor and gear faults in Section 5, the next stage of the analysis involves reducing the dimensionality of such features using PCA as well as examining the abilities of different combination of features to retain the highest variability. The implementation of PCA was based on the theories described in Equations (7)–(10). The four classes of features considered for this study are rotor/shaft only (α_1_–α_5_); GMFs only (β_1_–β_5_), combined rotor/shaft and GMFs (α_1_–α_5_) + (β_1_–β_5_) and SE-GMFs (γ_1_–γ_5_) features. The content distributions for 10 PCs were compared for all classes of features at all speeds as shown in Table 4. Since it is well established that the most significant information will usually reside within the first few PCs, the performance of combined PC_1–2_ and PC_1–3_ was compared, where it can be seen that PC_1–3_ held slightly superior information, which implies that it holds the potential to offer the most distinctive classification for all scenarios.

**Figure 7:**
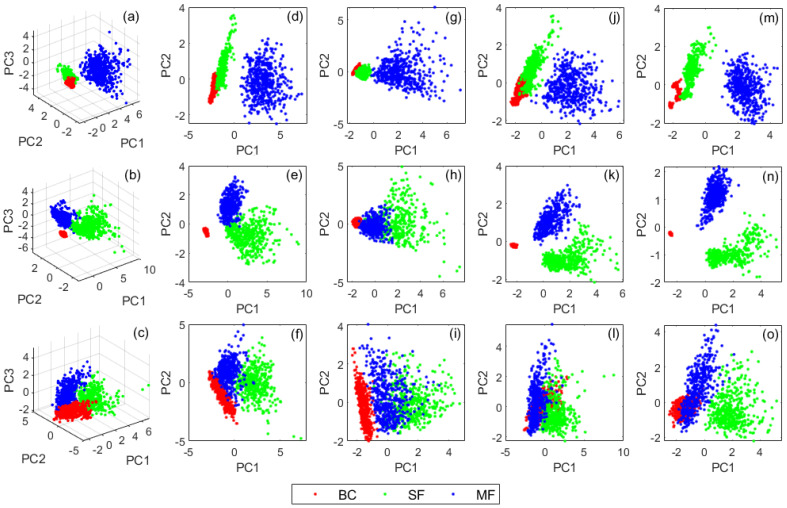
PCs combinations for all scenarios: (**a**–**c**) PC_1–3_ for (α_1_–α_5_) + (β_1_–β_5_) at 7, 14, and 21 Hz, respectively; (**d**–**f**) PC_1–2_ for (α_1_–α_5_) + (β_1_–β_5_) at 7, 14, and 21 Hz, respectively; (**g**–**i**) PC_1–2_ for (α_1_–α_5_) at 7, 14, and 21 Hz, respectively; (**j**–**l**) PC_1–2_ for (β_1_–β_5_) at 7, 14, and 21 Hz respectively; (**m**–**o**) PC_1–2_ for (γ_1_–γ_5_) at 7, 14, and 21 Hz, respectively.

However, owing to the higher data requirements for the PC_1–3_ combination and correspondingly higher computational burden, the performance of the PC_1–2_ combination was additionally explored for comparative purposes as shown in Figure 7. As anticipated, the PC_1–3_ combination offered the best separation between the clusters that represent all machine conditions (Figure 7a–c), but the performances of several PC_1–2_ combinations were also encouraging, especially those that involved (α1–α5) + (β1–β5) and SE-GMFs (γ_1_–γ_5_) features in Figure 7e,f and Figure 7m–o, respectively. However, despite the good intercluster separations achieved with PCA, its manual approach makes it unsustainable for routine diagnosis of rotating machines, whereby huge amounts of data related to highly dynamic scenarios is involved. Based on this perceived limitation, there is a need for applying approaches that possess self-learning capabilities with minimal human intervention. One of such approaches is ANN, whose proficiency with the current framework has already been established with several rotor-related faults at various machine speeds.

The current study aims to consolidate as well as extend the robustness of the approach by investigating an entirely novel class of faults with regards to a CCS-based data fusion approach. The classification problem is defined as classifying the data into 3 classes (BC, SF, and MF) based on the selected features. To achieve this, 3 ANN architectures were examined for PC_1–3_ and PC_1–2_ combinations for all cases at all speeds. For the PC_1–3_ combination, the ANN architectures had 3–10–3, 3–20–3, and 3–30–3 configurations for ANN_1_, ANN_2_, and ANN_3_ respectively. For PC_1-2_ combinations, however, 2–10–3, 2–20–3, and 2–30–3 configurations were respectively applied for ANN_1_, ANN_2_, and ANN_3_. In order to ascertain the performance without PCA, ANN_4_ was computed without PCA and its outcome was also used for comparison (i.e., 10–30–3 for (α_1_–α_5_) + (β_1_–β_5_) and 5–30–3 for (γ_1_–γ_5_)). It is vital to note that 3–10–3, 3–20–3, 3–30–3, 2–10–3, 2–20–3, 2–30–3, 10–30–3, and 5–30–3 for individual ANN configurations, respectively representing the inputs, number of neurons for hidden layers, and outputs. The analysis was conducted based on a 70–15–15 random split of features extracted from measured VM data for training, validation, and testing, respectively. The PCA step described in Section 3.2.1 was then applied to 85% of the datasets (i.e., combined training and validation datasets), after which 15% of the datasets were then extracted from the 85% and used for validation. Subsequently, the classification steps described in Section 3.2.2 were then applied to the testing datasets. The transfer function adopted here is the sigmoid symmetric transfer function. Since the ANN type is backward propagation, scaled conjugate gradient (SCG) was used as a learning algorithm as well as for overfitting avoidance. Table 5, Table 6 and Table 7 provide full details of the configurations and performance at all speeds.

**Figure 8:**
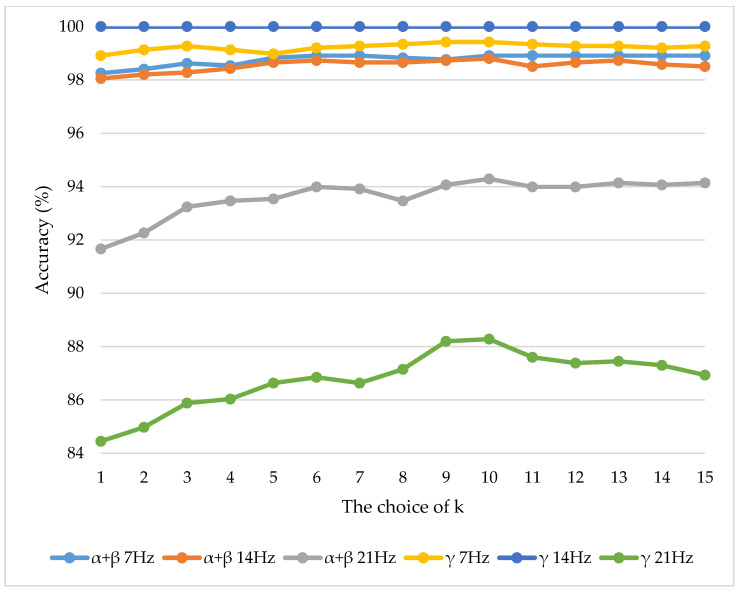
Classification accuracies for a range of k values of k-NN.

There are 2 aspects of evaluating the performance of ANNs: one is the accuracy of fitting and the other is whether overfitting occurs. As shown in Table 5, Table 6 and Table 7, the results of different ANN architectures are very similar for same scenarios (i.e., same speeds and same sets of features). For instance, at 21Hz, the accuracy of ANN with inputs of PC_1–3_ for (α_1_–α_5_) + (β_1_–β_5_) was significantly better than that of PC_1–2_ for (α_1_–α_5_) + (β_1_–β_5_) and PC_1–2_ for (γ_1_–γ_5_). However, PC_1–2_ for (γ_1_–γ_5_) has the best classification results at the other 2 speeds. The ANN computed based on inputs without PCA yielded similar results overall, except that it performed better than PC_1–2_ for both (α_1_–α_5_) + (β_1_–β_5_) and (γ_1_–γ_5_) at 21Hz. This was because the percentages of explained variance by PC_1–2_ at 21Hz were relatively small (i.e., 45.577 or 3% and 73.442 or 8%). In general, there was no significant difference in the accuracies of the ANNs trained based on these 3 features as inputs at the same speeds. Further evidence on the reason for not using (α_1_–α_5_) + (β_1_–β_5_) + (γ_1_–γ_5_) as a feature in this study are depicted in Table A1 within Appendix A. In order to demonstrate the rationale behind using ANN as the machine learning classifier in this study, the classification accuracy of ANN was compared to those obtained from three other machine learning classifiers, namely, k-NN (k = 10), naïve Bayes, and linear SVM as shown in Table 8. The comparisons were based on two input feature types. Figure 8 shows that k = 10 for k-NN had overall best results in a range of k from 1 to 15 for all considered scenarios. Therefore, k = 10 has been chosen for comparisons in Table 8. The results indicate that the ANN method outperformed all other classifiers for every scenario considered in this study.

In order to ensure good classification effects, overfitting must be avoided. Since the decision boundary of the classifier trained by the input sets with 3 dimensions or above is reasonably hyperplane in nature, it is difficult to visualise the decision rules in a 2-dimensional map. Thus, the difficulty of direct observation on whether there is an overfitting problem in an ANN with high-dimensional inputs could yield challenges in practice. On the contrary, the decision rules of ANNs trained by 2-dimensional input sets can be easily displayed. Based on this premise, it is fair to assume that 2-dimensional training input sets with PCA are advantageous when the variations in accuracy are minimal.

**Figure 9:**
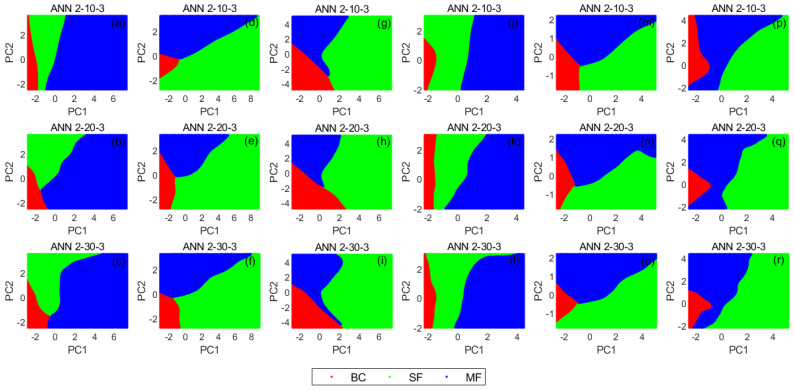
Visualised decision maps for ANN_1_–ANN_3_ using PC_1–2_ as inputs for all cases: (**a**–**c**) (α_1_–α_5_) + (β_1_–β_5_) at 7 Hz; (**d**–**f**) (α_1_–α_5_) + (β_1_–β_5_) at 14 Hz; (**g**–**i**) (α_1_–α_5_) + (β_1_–β_5_) at 21 Hz; (**j**–**l**) (γ_1_–γ_5_) at 7 Hz; (**m**–**o**) (γ_1_–γ_5_) at 14 Hz; (**p**–**r**) (γ_1_–γ_5_) at 21 Hz.

It is well known that overfitting is an immense threat to the abilities of machine learning algorithms to accurately detect and classify new data, owing to the incorporation of extrinsic details during the training process. In this study, it is envisaged that the application of SCG as a training algorithm will help mitigate potential problems. During individual trainings, the initial values of neurons will be reset randomly, with a corresponding random redivision of the data into 3 distinct groups for training, validation, and testing. This approach implies that training multiple times with a single input set will produce different results with slightly different decision boundaries. Figure 9 shows the decision rules of ANNs trained by PC_1–2_ for (α_1_–α_5_) + (β_1_–β_5_) and (γ_1_–γ_5_) at different speeds (i.e., typical results after a single round of training). The input datasets here correspond to (d–f) and (m–o) in Figure 7. The number of neurons of the hidden layer is considered as a variable for controlling potential overfitting problems. The emergence of complex boundary curves and narrow or slender envelope area within decision regions are likely indications of overfitting. For instance, the curvature of the decision boundary that exists between SF and MF regions is quite steep in Figure 9c as well as the visible elongated sharp strip area at the lower end of MF region in Figure 9i indicate that ANN_3_ could be associated with overfitting problems. With reference to Figure 9, decision maps generated from more neurons tend to be associated with overfitting problems. Therefore, since there are only 2 input values and 3 output classes in this classification, 10 neurons are adjudged sufficient for optimised, reasonably comprehensive, and complete classification of the cases considered in this study (i.e., increasing the number of neurons may not lead to better results). However, as more and more fault types emerge, it may be necessary to increase the number of neurons to boost the accuracy of classification. In general, the results presented here show that the initially proposed automated fault diagnosis framework is capable of identifying and classifying common gearbox faults using very simple and well-known features such as amplitude of rotor-related and GMF harmonics. This thereby provides good encouragement that the approach may be suitable for integrating rotor and gearbox fault diagnosis into a single framework in the near future.

## 7. Validation Dataset

In order to further examine the effectiveness of the applied method for classifying independent datasets, the study obtained publicly available gearbox fault datasets provided in an earlier study by Shao et al. [44] for validation. According to Shao et al. [44], the validation gearbox datasets were acquired from a drivetrain dynamic simulator, whereby two kinds of working conditions (i.e., rotating speed and load) were experimentally simulated. The rotating speed and load configurations were set to 20 Hz–0 V and 30 Hz–2 V. Vibration data were collected using 6 accelerometers mounted at 2 measuring positions. Position one (P1) datasets were acquired from the planetary gearbox measurement location in three directions (i.e., x, y, and z). Similarly, Position two (P2) datasets were acquired in three directions (i.e., x, y, and z) but from a parallel gearbox.

The different types of faults for both gearboxes are shown in Table 9. The datasets contain five different working conditions (i.e., four fault types and one healthy). Hence, the fault diagnosis here is based on a 5-class classification task. For each of the scenarios, 10 VM datasets were acquired for approximately 200 s. During spectrum and CCS calculation, the signal processing parameters used are 5120 Hz sampling frequency ($f_{s}$), 80% segment overlap, 0.5 Hz frequency resolution (df), 249 number of segment averages, 10,240 number of FT data points (N), and Hanning window. For CCS computation, two forms of data fusion approaches were considered. The former on the one hand was implemented to fuse the data from all six accelerometers mounted at the two measurement locations into a single spectrum (i.e., P1xyz+P2xyz). The latter on the other hand was implemented to fuse the data from the two accelerometers that had the same orientation (i.e., P1x+P2x, P1y+P2y, and P1z+P2z). ANN_1_ (2–10–5) was used as a classifier, and the PC_1–2_ of shaft harmonic features (α_1_–α_5_) was used as input. The analysis was also conducted based on a 70–15–15 random split of data for training, validation, and testing, respectively. Based on the linear space generated by the application of PCA to the training and validation datasets, linear transform was then implemented on the testing datasets.

**Figure 10:**
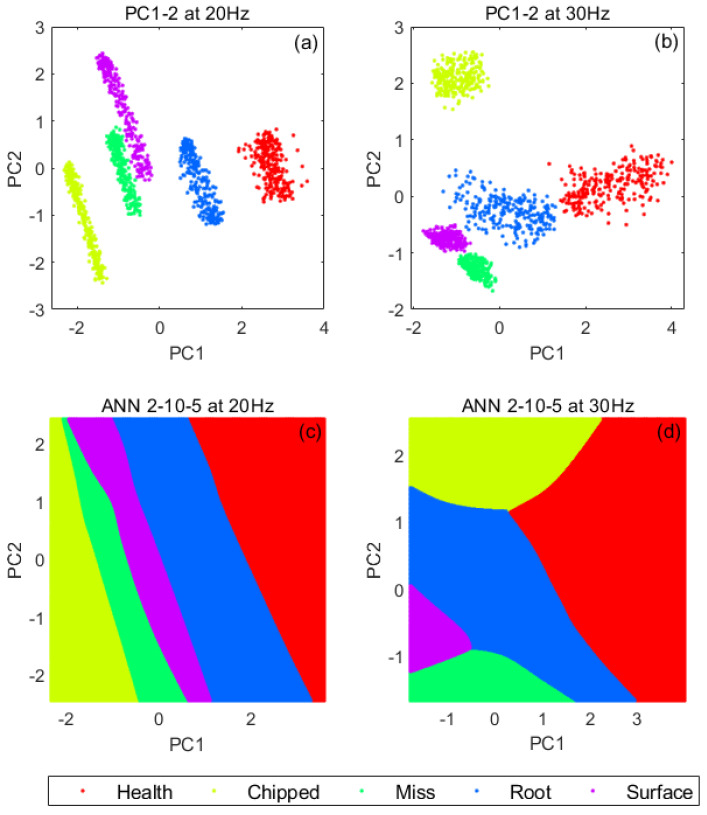
PC_1–2_ and visualised decision maps for ANN_1_ using PC_1–2_ as inputs for (α_1_–α_5_) of validation datasets at 20 and 30 Hz: (**a**) PC_1–2_ at 20 Hz; (**b**) PC_1–2_ at 30 Hz; (**c**) ANN_1_ at 20 Hz; (**d**) ANN_1_ at 30 Hz.

The classification problem is defined as classifying the data into 5 classes (Health, Chipped, Miss, Root, and Surface) based on the selected features. Table 10 and Table 11 and Figure 10 show the results of the validation, where it can be observed that the applied approach effectively classifies all the considered validation datasets, thereby confirming the robustness. It was also observed that the outcomes obtained by integrating all six accelerometers are better than when only two accelerometers were used.

## 8. Concluding Remarks and Future Possibilities

Industrial rotating machines are multi-component assets which imply that a truly holistic faults classification framework should be capable of detecting anomalies associated with each component, since faults rarely occur in isolation. Previous studies on CCS data fusion have effectively rationalised vibration-based condition monitoring data of rotating machines as well as characterised most of the faults commonly encountered in practice. However, as valuable as the findings from those studies were, their applications have been restricted to rotor-related faults such as misalignment, rub, crack, looseness, and bend, which often raises questions about the efficacy of the technique. Additionally, the founding works on CCS were based on manual classifications, which may be unrealistic for routine VM that often involves the analysis of huge amounts of data on a continuous basis. In order to alleviate these challenges, this study enhances current knowledge through the following two main premises:

- It establishes a framework by which the diagnosis of multiple classes of rotating machine faults can be automated through machine learning algorithms.
- It incorporates gearbox fault detection into a single framework. Hence, the extension provided here now considers an entirely different and unique class of rotating machine components—the gearbox, so as to complement earlier findings and ascertain robustness.

The results observed further affirmed the proficiency of the framework for both rotor and gearbox faults. Additionally, the proposed approach is primarily based on tools and features that are universally established across academia and industry (especially amplitude spectrum), thereby easing the transfer of theoretical knowledge into practice. Considering that all studies related to the application of CCS and pCCS data fusion approaches to fault diagnosis feature generation have been purely experimental, future endeavours will focus on validating such experimental scenarios within a theoretical environment.

## Figures and Tables

**Table 1:** Comparison between the proposed framework and the related state-of-the-art works.

Reference	Data Type	Classification Algorithm	Use of Data Fusion	Fault Classes Considered	Limitation
[38,39,40,41]	Vibration data	PCA and spectrum-based liner classification	Yes	Rotor faults	Classification approach used does not involve machine learning, thereby making the approach unable to self-learned from historical data. Additionally, all the simulated cases are rotor-related.
[42]	Vibration data	ANN, SVM, k-NN, naïve Bayes	Yes	Rotor faults	All the simulated cases are rotor-related.
[43]	Vibration data	CNN	No	Gears	Computational intensiveness due to the application of deep learning approach. This study only considered a single fault class. Hence, there might be need to further investigate the applicability on other faults.
[44]	Vibration data	CNN	No	Motor, gears and bearing faults	Although this study considered multiple fault classes, the approach is also computationally intensive and would lead to costlier solutions.
[45]	Current data	ANFIS	No	Gears	This study will benefit from the implementation of multi-sensor data fusion (such as electrical and mechanical data), so as to enhance the reliability of fault diagnosis.
[46]	Current data	CNN	Yes	Gears	Computational intensiveness due to the application of deep learning approach. This study only considered a single fault class. Hence, there might be a need to further investigate the applicability for other faults.
[47]	Vibration data	FICF	No	Gears and bearing faults	FICF is suitable for multi-sample training but the convolution activation limits its performance during single sample operations. Additionally, FICF is often considered a high-efficiency technique, but the poor noise adaptability sometimes undermines its proficiency.
Present study	Vibration data	ANN	Yes	Rotor and gear faults	The only limitation envisaged with the current approach is its initial requirement for training data for different fault types, which is also common to most of the aforementioned techniques.

**Table 2:** Technical specifications of main rig components.

Rig Component	Description (Abbreviation)	Specification/Dimension
Electric motor	Horsepower	2.0
	Speed	3600 RPM
	Maximum torque	3.9 Nm
	Type	Shunt
	Current	6.8 Amps
	Voltage	200 V
Shafts	Type	Mild steel
	Length	1 040 mm
	Diameter	35 mm
Gears	Type	Helical (key-mounted)
	Pitch circle diameter (PCD)	107 mm
	Face width	37 mm
	Circular thickness	4.5 mm
	Number of teeth	35
	Addendum	2.35 mm
	Dedendum	1.95 mm
Bearings	Type	Anti-friction ball bearings
	Make	SKF
	Model	SY20TF/RA SEY20/NP20
	Number of rolling elements	8
	Diameter of rolling elements	7.938 mm
	Bearing width	31 mm
	External diameter	47 mm
	Internal diameter	20 mm
	Bearing pitch circle diameter	33.5 mm
Pulleys	Type	Toothed, taper lock-mounted
	Thickness	32 mm
	Tooth thickness	6.8 mm
	Drive pulley diameter	100 mm
	Driven pulley diameter	125 mm
Belts	Type	Toothed, timing
	Model	Fenner 200H-100

**Table 3:** Technical specifications of main instruments.

VM Instrument	Parameter	Specification
Accelerometer	Model No.	352C33
	Sensitivity (±10%)	100 mV/g
	Frequency range (±5%)	0.5 to 10,000 Hz
	Resonant frequency	≥50 kHz
	Temperature range	−65 to +200 °F
	Settling time (within 10% of bias)	<10 s
Signal conditioner	Input sensor type	ICP, voltage, charge
	Voltage gain	×0.1 to ×200
	Voltage gain increment	0.1
	Charge conversion (selectable)	0.1, 1.0, 10.0 mV/pC
	Frequency range (gain <100)	0.05 to 100 kHz
	Frequency range (gain 100)	0.05 to 75 kHz
ADC	Number of channels	16 differential/32 single ended
	ADC resolution	16 bits
	Sampling rate	250 kS/s single channel; 250 kS/s multi-channel (aggregate)
	Input range	±10 V, ±5 V; ±1 V, ±0.2 V
	Input FIFO size	4095 samples

**Table 4:** Percentage of explained variance by each principal component (%).

Feature	(α_1_–α_5_) + (β_1_–β_5_)			(α_1_–α_5_)			(β_1_–β_5_)			(γ_1_–γ_5_)		
Frequency	7 Hz	14 Hz	21 Hz	7 Hz	14 Hz	21 Hz	7 Hz	14 Hz	21 Hz	7 Hz	14 Hz	21 Hz
PC_1_	62.6576	57.2374	29.9398	68.7087	55.7500	43.1111	67.6914	68.2912	38.7355	72.2149	75.0048	51.7278
PC_2_	7.8065	12.0692	15.6375	11.6652	14.0066	19.1375	13.8599	18.4893	22.1045	13.6757	17.8493	21.7150
PC_3_	6.4451	6.9354	10.9601	9.3631	12.0432	16.4981	9.7019	6.2872	16.7807	8.7733	3.1274	11.5762
PC_4_	5.5788	5.6463	9.5130	5.7620	9.8999	10.9578	4.9216	3.9276	13.0665	3.0254	2.4268	8.2653
PC_5_	4.5685	4.5533	8.1750	4.5010	8.3003	10.2954	3.8252	3.0047	9.3127	2.3107	1.5916	6.7156
PC_6_	4.0954	4.2480	6.3779	------	------	------	------	------	------	------	------	------
PC_7_	2.7094	3.2698	5.5352	------	------	------	------	------	------	------	------	------
PC_8_	2.2992	2.7340	5.1251	------	------	------	------	------	------	------	------	------
PC_9_	2.0604	1.8771	4.7339	------	------	------	------	------	------	------	------	------
PC_10_	1.7791	1.4295	4.0022	------	------	------	------	------	------	------	------	------
PC_1–2_	70.4641	69.3066	45.5773	80.3739	69.7566	62.2486	81.5513	86.7805	60.8400	85.8906	92.8541	73.4428
PC_1–3_	76.9092	76.2420	56.5374	89.7370	81.7998	78.7467	91.2532	93.0677	77.6207	94.6639	95.9815	85.0190

**Table 5:** ANN properties for (α_1_–α_5_) + (β_1_–β_5_) using PC_1–3_ and without PCA.

Parameters		ANN_1_			ANN_2_			ANN_3_			ANN_4_		
Network STRUCTURE		3–10–3			3–20–3			3–30–3			10–30–3 (without PCA)		
Rotation Frequency		7 Hz	14 Hz	21 Hz	7 Hz	14 Hz	21 Hz	7 Hz	14 Hz	21 Hz	7 Hz	14 Hz	21 Hz
**Accuracy (%)**	Training	99.1	98.8	98.2	99.1	99.0	98.2	99.3	99.3	98.5	98.9	100	98.7
	Validation	99.5	98.5	98.5	99.5	99.5	98.5	99.5	99.5	98.5	99.5	100	99.0
	Testing	99.5	99.0	98.5	99.5	99.5	99.0	99.5	100	99.0	98.5	100	97.0
	Overall	99.2	98.8	98.3	99.2	99.2	98.3	99.3	99.4	98.6	98.9	100	98.5

**Table 6:** ANN properties for (α_1_–α_5_) + (β_1_–β_5_) using PC_1–2_ and without PCA.

Parameters		ANN_1_			ANN_2_			ANN_3_			ANN_4_		
Network Structure		2–10–3			2–20–3			2–30–3			10–30–3 (without PCA)		
Rotation Frequency		7 Hz	14 Hz	21 Hz	7 Hz	14 Hz	21 Hz	7 Hz	14 Hz	21 Hz	7 Hz	14 Hz	21 Hz
**Accuracy (%)**	Training	98.9	99.1	95.1	98.9	99.1	95.3	98.9	99.1	95.4	98.9	100	98.7
	Validation	99.0	99.0	95.0	99.0	99.0	95.5	99.0	99.0	95.5	99.5	100	99.0
	Testing	99.0	99.0	95.0	99.0	99.0	94.5	99.5	99.0	93.5	98.5	100	97.0
	Overall	98.9	99.1	95.0	98.9	99.1	95.2	99.0	99.1	95.1	98.9	100	98.5

**Table 7:** ANN properties for (γ_1_–γ_5_) using PC_1–2_ and without PCA.

Parameters		ANN_1_			ANN_2_			ANN_3_			ANN_4_		
Network Structure		2–10–3			2–20–3			2–30–3			5–30–3 (without PCA)		
Rotation Frequency		7 Hz	14 Hz	21 Hz	7 Hz	14 Hz	21 Hz	7 Hz	14 Hz	21 Hz	7 Hz	14 Hz	21 Hz
**Accuracy (%)**	Training	99.4	100	88.2	99.5	100	89.3	99.5	100	89.3	99.5	100	97.5
	Validation	99.5	100	89.0	99.5	100	88.5	99.5	100	89.5	99.0	100	96.5
	Testing	99.5	100	89.0	99.5	100	90.5	99.5	100	87.5	99.5	100	97.0
	Overall	99.4	100	88.4	99.5	100	89.3	99.5	100	89.0	99.4	100	97.3

**Table 8:** Classifier properties for (α_1_–α_5_) + (β_1_–β_5_), and (γ_1_–γ_5_) using PC_1–2_.

Parameters	Features	Rotation Frequency	ANN_1_ (2–10–3)	k-NN (k = 10)	Naïve Bayes	Linear SVM
**Accuracy (%)**	(α_1_–α_5_) + (β_1_–β_5_)	7 Hz	98.9	98.9	98.5	98.8
		14 Hz	99.1	98.8	98.5	98.7
		21 Hz	95.0	94.3	91.5	94.3
	(γ_1_–γ_5_)	7 Hz	99.4	99.4	98.0	99.3
		14 Hz	100	100	99.7	100
		21 Hz	88.4	88.3	87.8	86.7

**Table 9:** Description of validation gearbox fault types [44].

Type	Description
Chipped	Crack occurs in the gear teeth
Miss	Missing one gear tooth
Root	Crack occurs in the root of the gear
Surface	Wear occurs in the surface of gear

**Table 10:** Percentage of explained variance by each principal component for (α_1_–α_5_) of public datasets (%).

Working Condition	20 Hz–0 V				30 Hz–2 V			
Measuring Position	P1xyz + P2xyz	P1x + P2x	P1y + P2y	P1z + P2z	P1xyz + P2xyz	P1x + P2x	P1y + P2y	P1z + P2z
PC_1_	54.9056	55.6459	41.0896	65.2457	42.4231	54.2842	39.2872	48.8888
PC_2_	19.5594	21.0863	24.5452	17.6613	27.5368	24.7716	23.0098	25.1579
PC_3_	13.8458	14.4407	19.5344	14.2051	13.1208	10.2944	17.9762	15.9588
PC_4_	10.4256	7.2607	13.3457	1.9847	9.8741	8.0938	11.5219	8.3389
PC_5_	1.2636	1.5664	1.4851	0.9032	7.0452	2.5560	8.2049	1.6557
PC_1–2_	74.4650	76.7322	65.6348	82.9070	69.9599	79.0558	62.2970	74.0467

**Table 11:** ANN_1_ (2–10–5) properties for (α_1_–α_5_) of public datasets using PC_1–2_.

Working Condition		20 Hz–0 V				30 Hz–2 V			
Measuring Position		P1xyz + P2xyz	P1x + P2x	P1y + P2y	P1z + P2z	P1xyz + P2xyz	P1x + P2x	P1y + P2y	P1z + P2z
**Accuracy (%)**	Training	100	100	91.5	100	100	99.5	87.5	96.7
	Validation	100	100	92.0	100	100	99.5	86.6	96.8
	Testing	100	100	90.4	99.5	100	99.5	86.6	95.2
	Overall	100	100	91.4	99.9	100	99.5	87.2	96.5

## Data Availability

Not applicable.

## Abbreviations

A	Matrix of the input data
$A_{CCS}$	Amplitude of composite coherent spectrum
$A_{SE}$	Spectral energy
$a_{ij}$	Element of the input data matrix A
${\bar{A}}_{j}$	Sample mean of elements of the jth column of A
B	Matrix of the new input data without labels
b	Total number of measurement points
$b^{\prime }$	Scalar bias term of artificial neural network
$b_{ij}$	Element of the new input data matrix B
$C_{X}$	Covariance matrix of X
df	Frequency resolution
$f_{GMF}$	Gear mesh frequency
$f_{k}$	Frequency
$f_{s}$	Sampling frequency
h	Ordinal number of data points
i	Ordinal number of rows of matrix
j	Ordinal number of columns of matrix
k	Number of nearest neighbours
L	Number of largest singular values
m	Number of samples
N	Number of data points
n	Number of features
$n_{R}$	Rotating speed of the pinion
$n_{s}$	Number of vibration signal segments
p	Ordinal number of measurement points
R	Real matrix
r	Ordinal number of vibration signal segments
S	Transformed score matrix
S	Coherent cross-power spectral density
$S_{CCS}$	Composite coherent spectrum
$S_{j}$	Sample standard deviation of the jth column of A
$S_{L}$	Transformed truncated score matrix
T	Score matrix for principal components analysis
$T_{L}$	Truncated score matrix
V	Orthogonal matrix
W	Weights of artificial neural network
X	Standardised matrix
X	Discrete Fourier transform
$X_{CCS}$	Component of composite coherent spectrum
$x_{ij}$	Element of the standardised matrix X
Y	Transformed matrix
$y_{ij}$	Element of the transformed matrix Y
z	Resultant class of machine health condition
$z_{t}$	Number of teeth on pinion
$\alpha_{1}$ $–\alpha_{5}$	Amplitudes of 1^st^–5^th^ harmonics of shaft speed
$\beta_{1}$ $–\beta_{5}$	Amplitudes of 1^st^–5^th^ harmonics of gear mesh frequency
$\gamma_{1}$ $–\gamma_{5}$	Spectrum energies of 1^st^–5^th^ harmonics of gear mesh frequency
$\gamma^{2}$	Coherence
Λ	Diagonal matrix

## Appendix A

As shown in the Table A1, the performance of (α_1_–α_5_) + (β_1_–β_5_) + (γ_1_–γ_5_) is very similar to that obtained from using only (γ_1_–γ_5_) at 7 and 14 Hz, but performed even more poorly at 21 Hz. This was adjudged to be owing to the extraction of both (β_1_–β_5_) and (γ_1_–γ_5_) from GMF information. Therefore, combining (γ_1_–γ_5_) with (α_1_–α_5_) + (β_1_–β_5_) would lead to information overload and possible redundancy, which would be counterintuitive to the primary aims of the paper.

**Table A1:** ANN_3_ properties for different features using PC_1–2_.

Feature		(α_1_–α_5_) + (β_1_–β_5_)			(γ_1_–γ_5_)			(α_1_–α_5_) + (β_1_–β_5_) + (γ_1_–γ_5_)		
Parameters		ANN_3_			ANN_3_			ANN_3_		
Network Structure		2–30–3			2–30–3			2–30–3		
Rotation Frequency		7 Hz	14 Hz	21 Hz	7 Hz	14 Hz	21 Hz	7 Hz	14 Hz	21 Hz
**Accuracy (%)**	Training	98.9	99.1	95.4	99.5	100	89.3	99.6	100	87.3
	Validation	99.0	99.0	95.5	99.5	100	89.5	99.5	100	87.0
	Testing	99.5	99.0	93.5	99.5	100	87.5	99.5	100	84.0
	Overall	99.0	99.1	95.1	99.5	100	89.0	99.6	100	86.8
